# Biosafety assessment of water samples from Wanzhou watershed of Yangtze Three Gorges Reservoir in the quiet season in *Caenorhabditis elegans*

**DOI:** 10.1038/s41598-018-32296-3

**Published:** 2018-09-20

**Authors:** Guosheng Xiao, Li Zhao, Qian Huang, Huihui Du, Dongqin Guo, Mingxing Xia, Guangman Li, Zongxiang Chen, Dayong Wang

**Affiliations:** 1 College of Biology and Food Engineering, Chongqing Three Gorges University, Wanzhou, 404100 China; 2 Medical School, Southeast University, Nanjing, 210009 China; 3 Wanzhou Entry-Exit Inspection and Quarantine Bureau, Wanzhou, 404100 China

## Abstract

We here employed a model animal of *Caenorhabditis elegans* to perform toxicity assessment of original surface water samples collected from Three Gorges Reservoir (TGR) in the quiet season in Wanzhou, Chongqing. Using some sublethal endpoints, including lifespan, body length, locomotion behavior, brood size, and intestinal reactive oxygen species (ROS) induction, we found that the examined five original surface water samples could not cause toxicity on wild-type nematodes. Nevertheless, the surface water sample collected from backwater area induced the significant increase in expressions of genes (*sod-2* and *sod-3*) encoding Mn-SODs in wild-type nematodes. Among the examined five original surface water samples, exposure to the original surface water sample collected from backwater area could further cause the toxicity in decreasing locomotion behavior and in inducing intestinal ROS production in *sod-3* mutant nematodes. Moreover, the solid phase of surface water sample collected from backwater area might mainly contribute to the observed toxicity in *sod-3* mutant nematodes. Our results are helpful for understanding the potential effects of surface water in the TGR region in the quiet season on environmental organisms.

## Introduction

Three Gorges Reservoir (TGR), the world’s largest reservoir, has a novel ecosystem in the upper stream of Yangtze River in China due to the formation of the largest and the longest yearly water-level drop^1,2^. In the recent years, with the development of industrialization and urbanization, both organic and inorganic pollutants may be released from the industrial and the residential wastewater and accumulated in the water or in the soils/sediments in the TGR region along Yangtze River^3–12^. Besides this, the spatial-temporal dynamics of bacterioplankton community and the suspended particulate matter have been detected in the TGR region^13–15^. Based on these possibilities, the ecological safety for the Yangtze River in the TGR region has received the great attention^16–18^.

Nematodes *Caenorhabditis elegans* is a classic model animal with the sensitivity to various environmental toxicants or stresses^19–24^. Based on the use of some sublethal endpoints including lifespan, development, reproduction, locomotion behavior, and oxidative stress, *C. elegans* has been proven to be valuable for *in vivo* toxicological assessment of environmental samples or toxicants^25–28^. Moreover, due to the property of model animal, *C. elegans* is helpful for elucidating the underlying cellular and molecular mechanisms for the observed toxicity of environmental toxicants^29–31^. Recently, the safety assessment of original surface water samples collected from TGR region in the flood season in Wanzhou, Chongqing has been performed using *C. elegans* as an assay animal model^32^. It has been shown that, after acute exposure, the original surface water sample collected from backwater area in the flood season could induce the obvious toxicity on wild-type nematodes^32^.

Besides the flood season, the quiet season is another important duration for the water in the TGR region, and most of the time (October-May) in TGR region in Wanzhou is in the quiet season. We hypothesize that some of the original surface water samples collected from TGR region in the quiet season may also have ecological risk under certain conditions. In this study, we further employed the animal model of *C. elegans* to perform the toxicity assessment on the original surface water samples collected from TGR region in the quiet season in Wanzhou, Chongqing. Our data suggest that original surface water sample collected from backwater area might have adverse effects on *sod-3* mutant nematodes. Additionally, the solid phase may contribute greatly to the observed toxicity of surface water sample collected from backwater area on *sod-3* mutant nematodes.

## Results

### Toxicity assessment of surface water samples in the TGR region in the quiet season in wild-type nematodes

**Figure 1:**
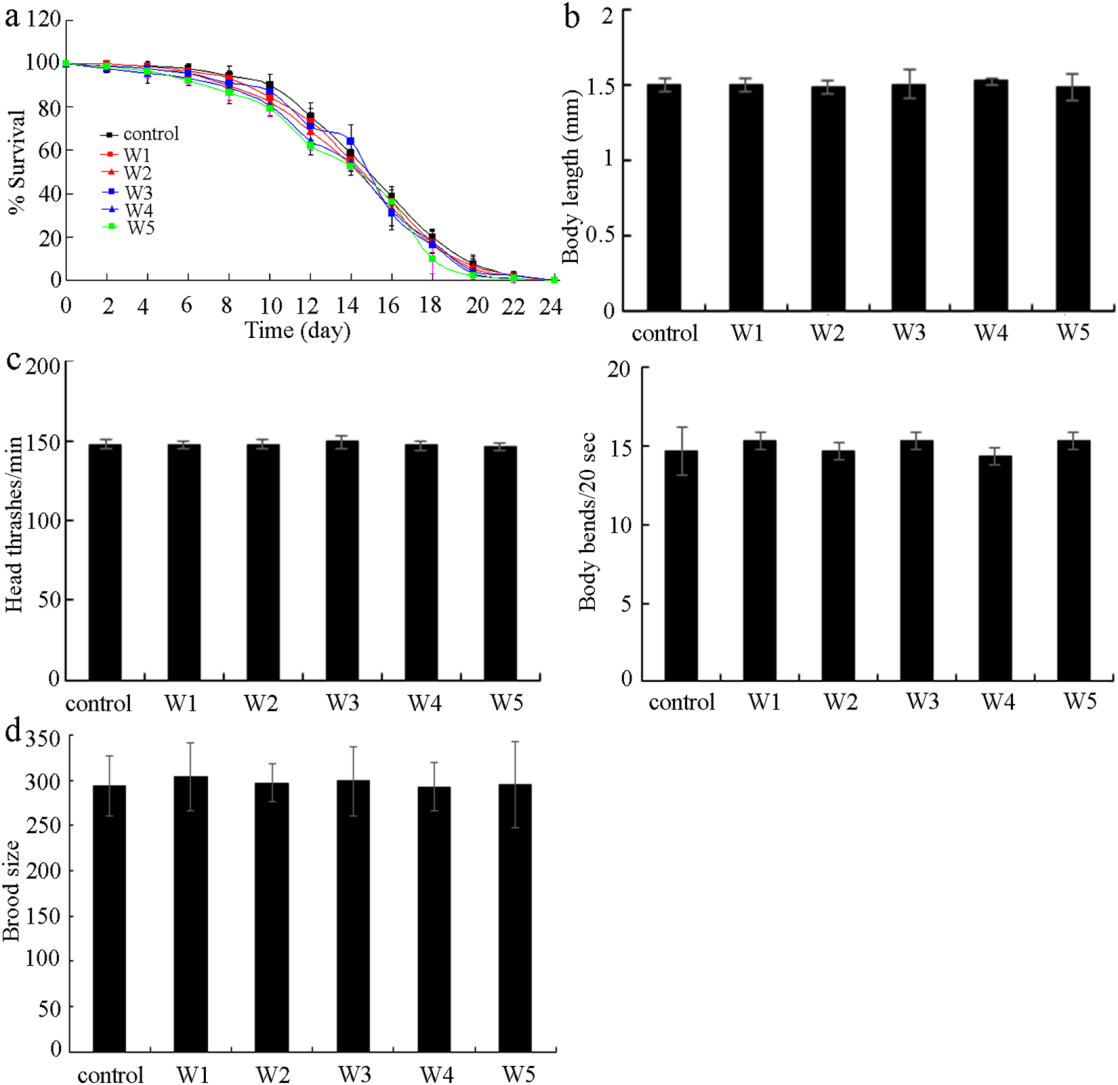
Effects of different surface water samples in the TGR region in the quiet season on lifespan (**a**), body length (**b**), locomotion behavior (**c**), and brood size (**d**) in wild-type nematodes. Exposures were performed from L4-larvae for 24-h. The differences between groups were analyzed using analysis of variance (ANOVA). The survival curve data were statistically analyzed using the log-rank test. Bars represent means ± SD.

We first employed the endpoints of lifespan, body length, locomotion behavior, and brood size to perform the toxicity assessment of five surface water samples (W1, W2, W3, W4, and W5) in the TGR region in the quiet season in wild-type nematodes. After acute exposure from L4-larvae for 24-h, surface water samples of W1, W2, W3, W4, and W5 all did not significantly affect the lifespan, the body length, the locomotion behavior as reflected by the head thrash and the body bend, and the brood size in wild-type nematodes (Fig. 1).

### Effect of surface water samples in the TGR region in the quiet season on induction of intestinal reactive oxygen species (ROS) production in wild-type nematodes

**Figure 2:**
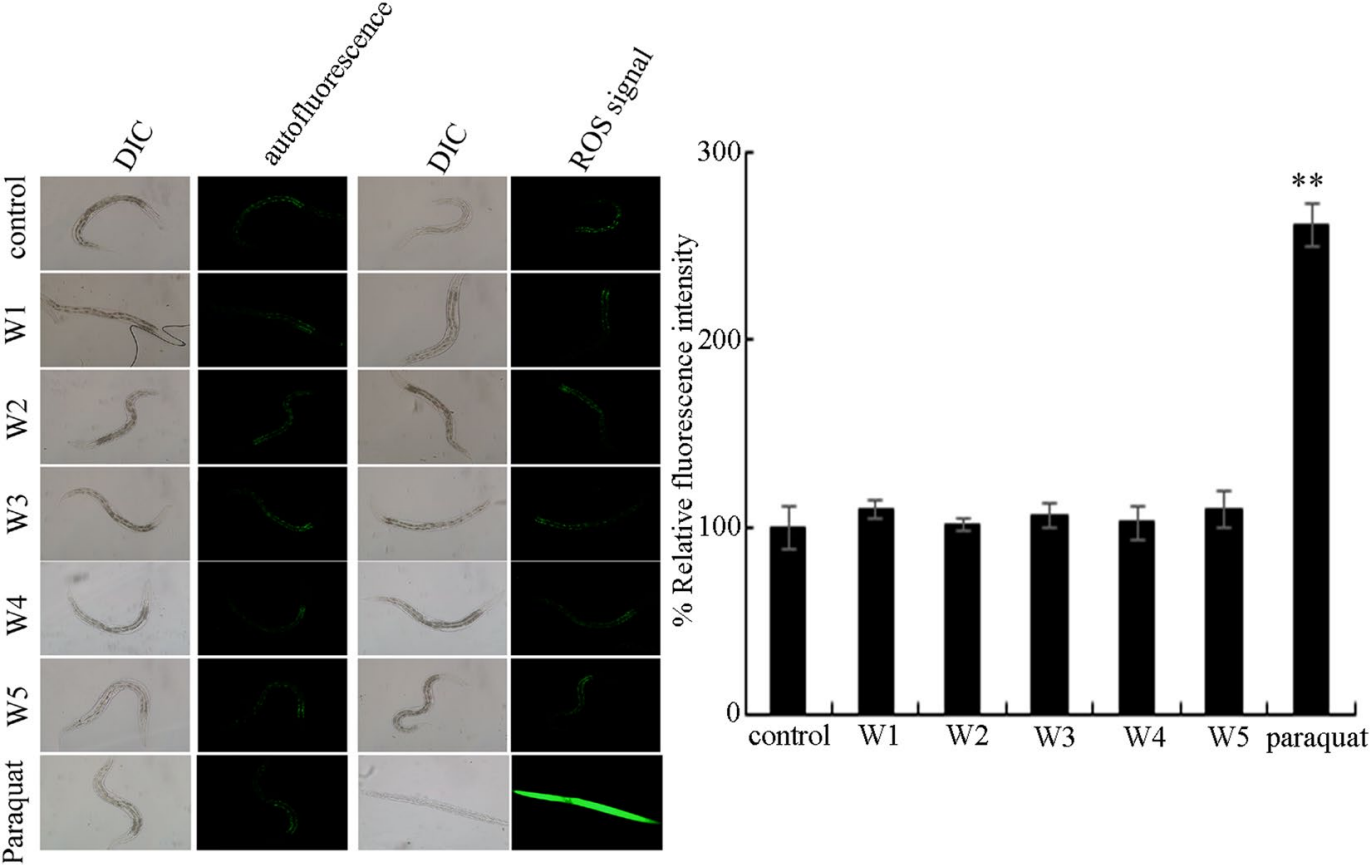
Effect of different surface water samples in the TGR region in the quiet season on induction intestinal ROS production in wild-type nematodes. Paraquat (2 mM), positive control. Exposures were performed from L4-larvae for 24-h. The differences between groups were analyzed using analysis of variance (ANOVA). Bars represent means ± SD. ^**^P < 0.01 *vs* control.

Considering the crucial role of oxidative stress in the toxicity induction of environmental toxicants or stresses^33–35^, we further determined the possible effect of surface water samples in the TGR region in the quiet season on wild-type nematodes. After acute exposure from L4-larvae for 24-h, the surface water samples of W1, W2, W3, W4, and W5 all could not induce the obvious intestinal ROS production in wild-type nematodes (Fig. 2).

### Effect of surface water samples in the TGR region in the quiet season on molecular basis for oxidative stress in wild-type nematodes

**Figure 3:**
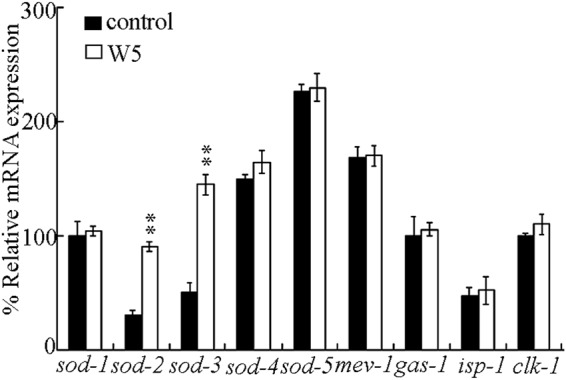
Effect of surface water sample of W5 on transcriptional expressions of *sod-1-5*, *mev-1*, *gas-1*, *isp-1*, and *clk-1* in wild-type nematodes. Relative quantification of the examined genes in comparison to reference *tba-1*. Exposures were performed from L4-larvae for 24-h. The differences between groups were analyzed using analysis of variance (ANOVA). Bars represent means ± SD. ^**^P < 0.01 *vs* control.

In nematodes, the superoxide dismutases (SODs) provide the antioxidative systems, and the proteins of MEV-1, GAS-1, ISP-1, and CLK-1 are components in mitochondrial complex or electron transport chain. The functions of these proteins act as the core molecular mechanisms of induction of oxidative stress in nematodes^36–42^. Although we did not observe the abnormal phenotypes, we still wonder whether certain molecular response associated with the oxidative stress could be activated in wild-type nematodes exposed to the examined surface water samples. Acute exposure to the surface water sample of W1, W2, W3, or W4 did not significantly alter the transcriptional expressions of all these examined genes in wild-type nematodes (data not shown). Acute exposure to the surface water sample of W5 also could not significantly affect the transcriptional expressions of *sod-1*, *sod-4*, *sod-5*, *mev-1*, *gas-1*, *isp-1*, and *clk-1* in wild-type nematodes (Fig. 3). Different from these, acute exposure to the surface water sample of W5 significantly increased the transcriptional expressions of *sod-2* and *sod-3* in wild-type nematodes (Fig. 3).

### Effect of *sod-3* mutation on potential toxicity of surface water samples in nematodes

**Figure 4:**
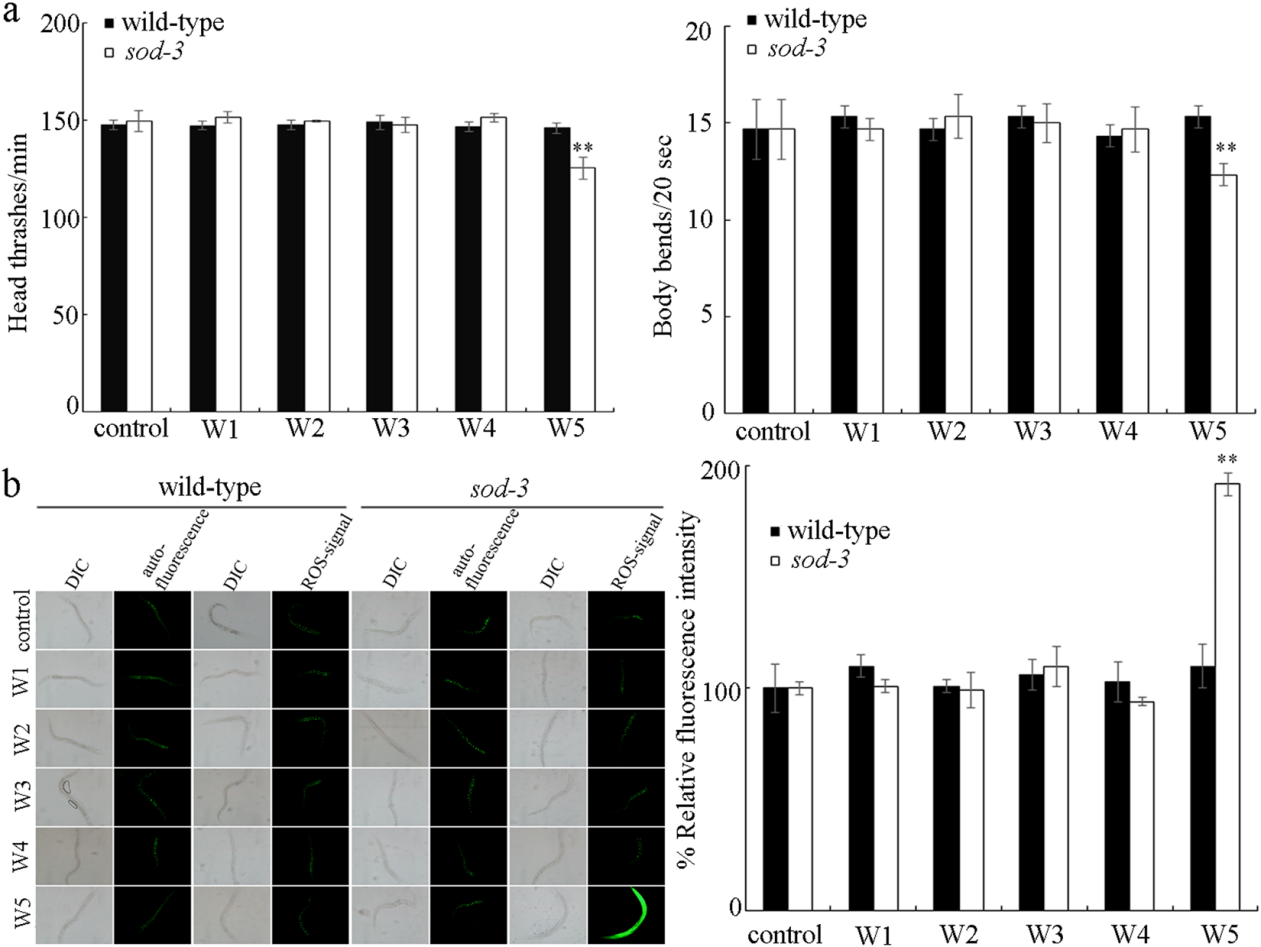
Effect of *sod-3* mutation on potential toxicity of surface water samples in the TGR region in the quiet season in decreasing locomotion behavior (**a**) and in inducing intestinal ROS production (**b**). Exposures were performed from L4-larvae for 24-h. The differences between groups were analyzed using analysis of variance (ANOVA). Bars represent means ± SD. ^**^P < 0.01 *vs* control.

In nematodes, *sod-2* and *sod-3* encode mitochondiral Mn-SODs. Mutation of any of these genes can induce a susceptibility to toxicity of environmental toxicants^33,43^. We further examined the possible toxicity of different surface water samples in *sod-3* mutant nematodes. We used locomotion behavior and induction of intestinal ROS production as the toxicity assessment endpoints. Even in the *sod-3* mutant nematodes, acute exposure to the surface water sample of W1, W2, W3, or W4 still could not cause the significant decrease in locomotion behavior and induction of intestinal ROS production (Fig. 4). In contrast, acute exposure to the surface water sample of W5 resulted in the significant decrease in locomotion behavior and induction of intestinal ROS production in *sod-3* mutant nematodes (Fig. 4).

### Analysis on the chemical pollutants from the sample W5

**Figure 5:**
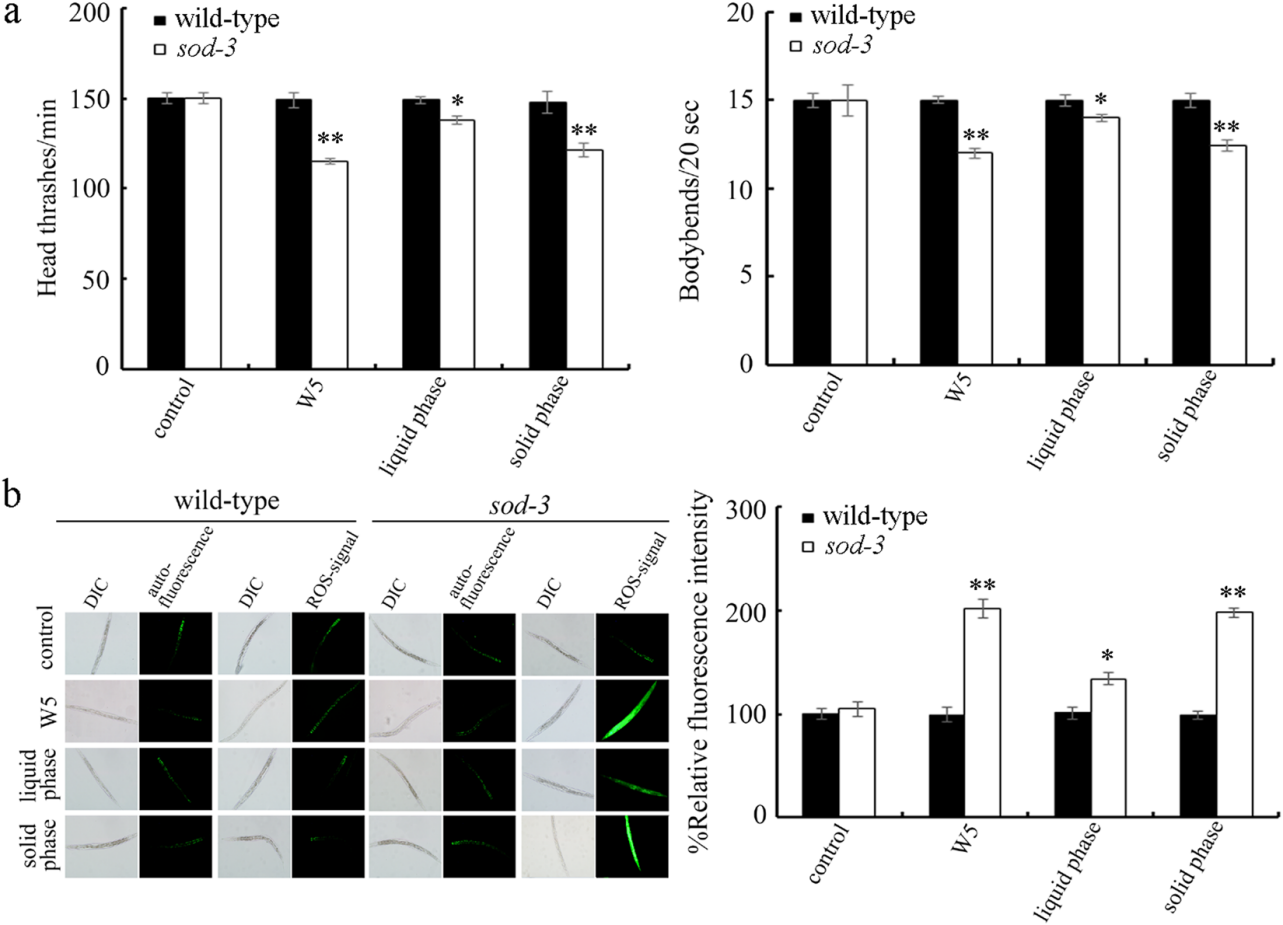
Effect of liquid phase or solid phase of the sample of W5 on locomotion behavior (**a**) and induction of intestinal ROS production (**b**) in *sod-3* mutant nematodes. Exposures were performed from L4-larvae for 24-h. The differences between groups were analyzed using analysis of variance (ANOVA). Bars represent means ± SD. ^*^P < 0.05 *vs* control, ^**^P < 0.01 *vs* control.

To determine the possible contributors from the surface water sample of W5 in inducing toxicity on *sod-3* mutant nematodes, both liquid phase and solid phase from the surface water sample of W5 were isolated by centrifugation at 10000 g for 10-min. After the centrifugation, the pellet was re-suspended with the equal volume of K medium to obtain the solution for solid phase, and the liquid phase existed in the supernatant. After acute exposure, the solid phase for surface water sample of W5 could lead to the obvious toxicity in decreasing locomotion behavior and in inducing intestinal ROS production in *sod-3* mutant nematodes (Fig. 5). In contrast, the liquid phase for surface water sample of W5 could only induce the moderate toxicity in decreasing locomotion behavior and in inducing intestinal ROS production in *sod-3* mutant nematodes (Fig. 5).

## Discussion

In this study, we first investigated the possible effects of five original surface water samples (W1, W2, W3, W4, and W5) in the TGR region in the quiet season on wild-type nematodes. With the aid of some sublethal endpoints (lifespan, body length, locomotion behavior, brood size, and intestinal ROS production), acute exposure to all these examined surface water samples in the TGR region in the quiet season did not cause the obvious toxicity on wild-type nematodes (Figs 1 and 2). This observation is very different from the effects of surface water samples in the TGR region in the flood season on wild-type nematodes. In wild-type nematodes, it has been shown that acute exposure to the surface water sample collected from the backwater area (W5) in the flood season could cause the significant decrease in locomotion behavior and induction of intestinal ROS production, although exposure to other four surface water samples in the flood season could not cause obvious toxicity^32^. These observations imply that, at least for the site of backwater area, the noticeable difference for the potential effect between surface water in the flood season and surface water in the quiet season may exist on environmental organisms. Additionally, our data also implies that, in the flood season, more chemical pollutants might be washed off into the TGR region than that in quiet season.

Although the surface water sample of W5 collected in the quiet season could not result in the obvious toxicity in wild-type nematodes, we found that exposure to this surface water sample could alter the molecular basis for oxidative stress to a certain degree. Exposure to surface water sample of W5 significantly increased the transcriptional expressions of genes (*sod-2* and *sod-3*) encoding mitochondrial Mn-SODs (Fig. 3). This observation implies one important possibility. That is, in the nematodes exposed to the surface water sample of W5, the potential toxicity of surface water sample of W5 could already induce a protection response mediated by the increased Mn-SODs, although the toxicity of surface water sample of W5 was still not enough to induce the obvious alterations in various phenotypes.

In this study, we further employed the *sod-3* mutant with the susceptible property to environmental toxicants to assess the potential toxicity of five surface water samples collected in the TGR region in the quiet season. We found that exposure to the surface water sample of W5 caused the significant decrease in locomotion behavior and induction of intestinal ROS production in *sod-3* mutant nematodes (Fig. 4). That is, mutation of *sod-3* may amplify the potential toxicity of the surface water sample of W5, which allows us to detect the obvious alteration in toxicity assessment endpoints, such as locomotion behavior and intestinal ROS production.

For the surface water sample of W5, our results imply that its solid phase may contribute greatly to the observed toxicity in *sod-3* mutant nematodes (Fig. 5). In contrast, the contribution of liquid phase for the surface water sample of W5 to its toxicity formation might be limited. The elemental analysis did not show obvious differences for the metals among the examined five surface water samples (Table S1), which implies that the observed moderate toxicity in the liquid phase for the surface water sample of W5 may be largely due to the organic pollutants. For the components existed in the solid phase in water in the TGR region, previous studies have implied the important roles of bacterioplankton community and suspended particulate matter^13–15^. Especially, previous study has detected the high prevalence and concentrations of pathogens and already identified some pathogens in the corresponding backwater area^44^. The detailed biological effects of identified pathogens on environmental organisms still need to be further examined. In the area for surface water sample of W5, we also observed a large amount of microplastics. For the technical limitations, it still unclear for the potential effects of these observed microplastics in the solid phase on environmental organisms. The assessment on the contribution of bacterioplankton community and suspended particulate matter to the toxicity formation of surface water sample of W5 in the TGR region in the quiet season is suggested to be further performed.

In conclusion, we examined the effects of five original surface water samples in the TGR region in the quiet season on nematodes. Using some sublethal endpoints, we found that all the examined original surface water samples in the TGR region in the quiet season could not cause the toxicity on wild-type nematodes. Nevertheless, acute exposure to the surface water sample of W5 induced the significant increase in transcriptional expressions of genes encoding Mn-SODs in wild-type nematodes. Moreover, mutation of *sod-3* resulted in the susceptibility to potential toxicity of the surface water sample of W5. Our data further imply that the solid phase of the surface water sample of W5 might mainly contribute to the observed toxicity in *sod-3* mutant nematodes. Our data will be helpful for our understanding the potential effects of surface water in the TGR region in the quiet season on environmental organisms.

## Methods

### Sample collection

**Figure 6:**
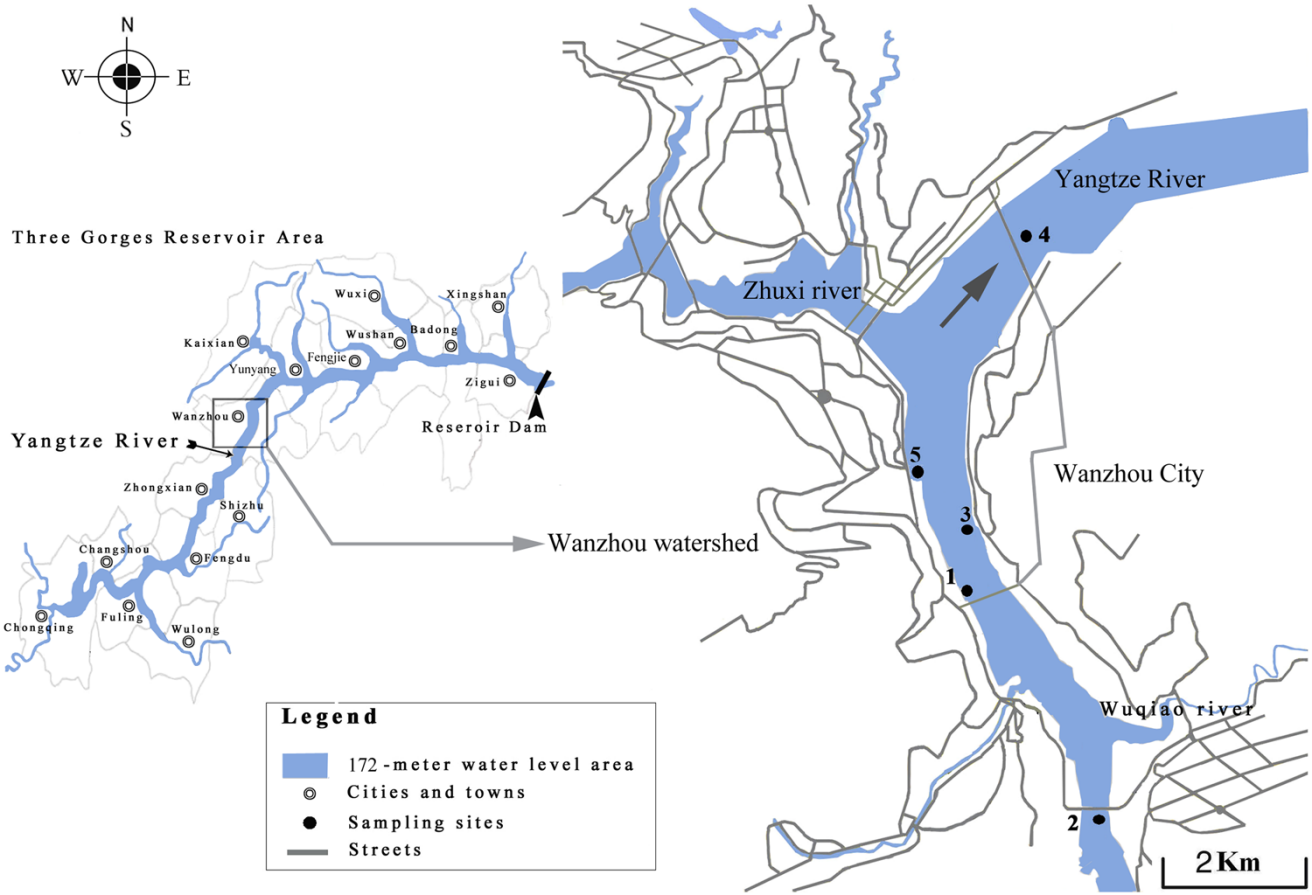
Sampling sites in the Three Gorges Reservoir.

The surface water samples were from 5 sampling sites (W1, shore water; W2, upstream; W3, shore water; W4, downstream; and W5, backwater area) in Wanzhou, Chongqing (Fig. 6), which are the same as those analyzed in our previous study^32^. The sampling season was selected in the quiet season (December 15, 2017). Water samples were collected according to the description of a standard method^45^. Surface water samples were collected at a depth of 0.5 m using a TN-S water sampler (Jintan Taina Instrument Factory, Jiangshu, China). Samples collected were stored in a car refrigerator (0 °C), and analyzed after being transported back to the laboratory in a cooler.

Water temperature, pH value, total dissolved solids, and turbidity were analyzed as described by Xiao *et al*.^32^. Information for daily flow and water level of the sampling sites was from Yangtze River Hydrological Network (http://www.cjh.com.cn). Major elements of surface water samples were determined by inductively coupled plasma atomic emission spectroscopy (ICP, GE Co., USA), and provided in Table S1. The related information for the collected surface water samples in TGR region is provided in Table S2.

### Animal maintenance and exposure

Wild-type N2 and mutant of *sod-3(gk235)* were used in this study. The animals were maintained on nematode growth medium (NGM) plates with *Escherichia coli* OP50 as a food source as described by Brenner^46^. Age synchronous L4-larvae were prepared by lysis of the hermaphrodite adults with bleaching mixture (0.45 M NaOH, 2% HOCl) in order to separate the eggs and the animals. Exposures were performed from L4-larvae for 24-h (acute exposure) in liquid solutions in the presence of food at 20 °C. Approximately 4 × 10^6^ colony-forming units (CFUs) of OP50 were added into the exposure solutions. The examined L4-larvae nematodes were directed transferred into the exposure solutions using the picker. Control animals were treated with liquid K medium.

### Toxicity assessment

After the exposure, the toxicity assessment was performed using lifespan, body length, brood size, locomotion behavior, and intestinal ROS production as the toxicity assessment endpoints.

Lifespan, an endpoint to reflect long-term effect of certain environmental toxicants^47^, was performed as described by Zhi *et al*.^48^. To examine the lifespan, the examined nematodes were transferred daily for the first 7 days of adulthood using a picker, and checked the survival every two-day. Nematodes will be scored as dead if they can not move even after repeated taps with a picker. Fifty nematodes were examined per treatment. Graphs are representative of three trials. The survival curve data were statistically analyzed using the log-rank test.

Body length was used to assess the growth of nematodes^32^. Body length was determined by measuring the flat surface length of nematodes using Image-Pro^®^ Express software. Thirty nematodes were examined per treatment.

Head thrash and body bend, endpoints to reflect the locomotion behavior^49,50^, were performed as described by Chen *et al*.^51^. After the exposure, the nematodes were first allowed to crawl freely for 1-min. Head thrashes were scored as the number of wavelengths the nematodes moved in a 1-min interval. Body bends were scored by eye for the number generated in a 20 s time interval. A change in the direction of propagation of the part corresponding to the posterior bulb of pharynx is defined as a body bend. Fifty nematodes were examined per treatment.

Brood size, an endpoint to reflect the reproductive capacity^52^, was performed as described by Zhao *et al*.^53^. The number of offspring at all stages beyond the egg was counted. Thirty nematodes were examined per treatment.

Intestinal ROS production, the endpoint to reflect the activation of oxidative stress^54^, was performed as described by Yang *et al*.^55^. The 5′,6′-chloromethyl-2′,7′dichlorodihydro-fluorescein diacetate (CM-H_2_DCFDA) can detect the presence of intracellular produced ROS species. The examined nematodes were labeled with CM-H_2_DCFDA (1 μM) for 3 h at 20 °C in the dark. After the labeling, the nematodes were analyzed at 488 nm of excitation wavelength and 510 nm of emission filter under a laser scanning confocal microscope. The semiquantified ROS was expressed as relative fluorescence units (RFU) and normalized to the autofluorescence. Thirty nematodes were examined per treatment. For intestinal ROS production assay, exposure to paraquat (2 mM), a ROS generator, was used as a positive control.

### Reverse-transcription and quantitative real-time polymerase chain reaction (qRT-PCR)

Total RNA of nematodes was isolated using Trizol (Invitrogen, UK) according to the manufacturer’s protocols. After cDNA synthesis, relative transcriptional expressions of certain genes were determined by real-time PCR in an ABI 7500 real-time PCR system with Evagreen (Biotium, USA). All reactions were performed in triplicate with the same cDNA samples. Relative quantification of the examined genes in comparison to reference *tba-1* gene encoding a tubulin protein was determined. The primer information was shown in Table S3.

### Statistical analysis

Data in this study were expressed as means ± standard deviation (SD). Statistical analysis using SPSS 12.0 software (SPSS Inc., Chicago, USA) and differences between groups using analysis of variance (ANOVA) were analyzed. Probability levels of 0.05 and 0.01 were considered to be statistically significant.

## Electronic supplementary material

Supporting Information
