# Supplementary material for: Biosafety assessment of water samples from Wanzhou watershed of Yangtze Three Gorges Reservoir in the quiet season in Caenorhabditis elegans
Source: Sci Rep. 2018 Sep 20;8:14102. doi: 10.1038/s41598-018-32296-3 (PMC6148280; doi:10.1038/s41598-018-32296-3)
Supplement: Supplementary file 1 — Supporting Information [file 41598_2018_32296_MOESM1_ESM.pdf]

**Biosafety assessment of water samples from Wanzhou watershed of Yangtze Three Gorges Reservoir in the quiet season in *Caenorhabditis elegans***

Guosheng Xiao[1], Li Zhao[2], Qian Huang[1], Huihui Du[1], Dongqin Guo[1], Mingxing Xia[3], Guangman Li[3], Zongxiang Chen[3] & Dayong Wang[2, *]

[1]College of Biology and Food Engineering, Chongqing Three Gorges University, Wanzhou 404100, China

[2]Medical School, Southeast University, Nanjing 210009, China

[3]Wanzhou Entry-Exit Inspection and Quarantine Bureau， Wanzhou 404100, China

[*]Corresponding author. E-mail address: dayongw@seu.edu.cn (D. Wang).

**Supporting Information:**

**Table S1.** Major elemental analysis in the collected surface water samples

| | W1 | W2 | W3 | W4 | W5 |
|---|---|---|---|---|---|
| Cd (μg/L) | ND | ND | ND | ND | ND |
| Pb (μg/L) | 0.3016 | 0.4123 | 0.3656 | 0.3272 | 0.3945 |
| Hg (μg/L) | 0.017 | 0.017 | 0.014 | 0.013 | 0.012 |
| As (μg/L) | 0.6 | 0.522 | 0.486 | 0.542 | 0.697 |
| Zn (μg/L) | ND | ND | ND | ND | ND |
| Cu (μg/L) | ND | ND | ND | ND | ND |
| Mn (μg/L) | ND | ND | ND | ND | ND |
| Cr (μg/L) | ND | ND | ND | ND | ND |
| Fe (mg/L) | 0.172 | 0.156 | 0.178 | 0.174 | 0.242 |
| K (mg/L) | 2.376 | 2.418 | 2.458 | 2.397 | 2.501 |
| Mg (mg/L) | 6.344 | 6.411 | 6.455 | 6.391 | 6.418 |

ND, not detectable.

**Table S2.** Information for the collected surface water samples in TGR region in the quiet season

| | W1 | W2 | W3 | W4 | W5 |
|---|---|---|---|---|---|
| Water level (m) | 172.9 | 172.9 | 172.9 | 172.9 | 172.9 |
| pH value | 8.012 | 8.103 | 8.107 | 8.098 | 8.087 |
| Longitude for sampling site | 108°23'36" | 108°25'15" | 108°25'15" | 108°24'2" | 108°23'25" |
| Latitude for sampling site | 30°47'30" | 30°45'39" | 30°48'30" | 30°49'22" | 30°47'45" |
| Altitude (m) | 160 | 170 | 150 | 150 | 150 |
| Water temperature (°C) | 16 | 16 | 15.8 | 16.3 | 15.3 |
| Turbidity (NTU) | 3.8 | 3.8 | 3.6 | 3.4 | 4.1 |
| Total dissolved solids (ppm) | 201 | 197.3 | 203.3 | 194.3 | 202 |

**Table S3.** Primers used for quantitative real-time polymerase chain reaction.

| Gene | Forward primer | Reverse primer |
|---|---|---|
| *tba-1* | TCAACACTGCCATCGCCGCC | TCCAAGCGAGACCAGGCTTCAG |
| *clk-1* | CACATACTGCTGCTTCTCGT | TGAACCAACAGATGAACCTT |
| *gas-1* | CTTGGTCTTTGGCTGTTGA | CTTGGTCTTTGGCTGTTGA |
| *isp-1* | GCAGAAAGATGAATGGTCC | CAGAAGCGTCGTAGTGAGA |
| *mev-1* | GGAATTCGCTTCTTAGGAT | GCAGTCTTGTTGCTCTTGT |
| *sod-1* | ACGCTCGTCACGCTTTAC | TCTTCTGCCTTGTCTCCG |
| *sod-2* | GGCATCAACTGTCGCTGT | ACAAGTCCAGTTGTTGCC |
| *sod-3* | TGACATCACTATTGCGGT | GGGACCATTCCTTCCAAA |
| *sod-4* | CACCAGATGACTCGAACA | AATGAGGCAAGAGAGTCG |
| *sod-5* | AAAGTAGAGTCGAAACGTGCTG | TGAAGTCCTGGTGACAATCCCT |
